# The Role of Fatty Acid Signaling in Islet Beta-Cell Adaptation to Normal Pregnancy

**DOI:** 10.3389/fendo.2021.799081

**Published:** 2022-01-05

**Authors:** Jee-Hye Kim, Viviane Delghingaro-Augusto, Jeng Yie Chan, D. Ross Laybutt, Joseph Proietto, Christopher J. Nolan

**Affiliations:** ^1^ Australian National University Medical School, Australian National University, Canberra, ACT, Australia; ^2^ Department of Immunology and Infectious Disease, John Curtin School of Medical Research, Australian National University, Canberra, ACT, Australia; ^3^ Garvan Institute of Medical Research, St Vincent’s Clinical School, University of New South Wales (UNSW), Sydney, NSW, Australia; ^4^ Department of Medicine (Austin Health), University of Melbourne, Heidelberg Heights, VIC, Australia; ^5^ Department of Endocrinology, The Canberra Hospital, Garran, ACT, Australia

**Keywords:** fatty acid signaling, fatty acid metabolism, insulin secretion, pancreatic islet beta-cell, hyperlipidemia, Ppargc1a, pregnancy, Sprague-Dawley rat

## Abstract

**Background:**

Maintenance of a normal fetal nutrient supply requires major adaptations in maternal metabolic physiology, including of the islet beta-cell. The role of lipid signaling processes in the mechanisms of islet beta-cell adaptation to pregnancy has been minimally investigated.

**Objective:**

To determine the effects of pregnancy on islet fatty acid (FA) metabolic partitioning and FA augmentation of glucose-stimulated insulin secretion (GSIS).

**Methods:**

Age matched virgin, early pregnant (gestational day-11, G11) and late pregnant (G19) Sprague-Dawley rats were studied. Fasted and fed state biochemistry, oral glucose tolerance tests (OGTT), and fasted and post-OGTT liver glycogen, were determined to assess *in vivo* metabolic characteristics. In isolated islets, FA (BSA-bound palmitate 0.25 mmol/l) augmentation of GSIS, FA partitioning into esterification and oxidation processes using metabolic tracer techniques, lipolysis by glycerol release, triacylglycerols (TG) content, and the expression of key beta-cell genes were determined.

**Results:**

Plasma glucose in pregnancy was lower, including during the OGTT (glucose area under the curve 0-120 min (AUC_0-120_); 655±24 versus 849±13 mmol.l^-1^.min; G19 *vs* virgin; *P*<0.0001), with plasma insulin concentrations equivalent to those of virgin rats (insulin AUC_0-120_; 97±7 versus 83±7 ng.ml^-1^.min; G19 vs virgin; not significant). Liver glycogen was depleted in fasted G19 rats with full recovery after oral glucose. Serum TG increased during pregnancy (4.4±0.4, 6.7±0.5; 17.1±1.5 mmol/l; virgin, G11, G19, *P*<0.0001), and islet TG content decreased (147±42, 172±27, 73±13 ng/µg protein; virgin, G11, G19; *P*<0.01). GSIS in isolated islets was increased in G19 compared to virgin rats, and this effect was augmented in the presence of FA. FA esterification into phospholipids, monoacylglycerols and TG were increased, whereas FA oxidation was reduced, in islets of pregnant compared to virgin rats, with variable effects on lipolysis dependent on gestational age. Expression of *Ppargc1a*, a key regulator of mitochondrial metabolism, was reduced by 51% in G11 and 64% in G19 pregnant rat islets compared to virgin rat islets (*P*<0.001).

**Conclusion:**

A lowered set-point for islet and hepatic glucose homeostasis in the pregnant rat has been confirmed. Islet adaptation to pregnancy includes increased FA esterification, reduced FA oxidation, and enhanced FA augmentation of glucose-stimulated insulin secretion.

## Introduction

Adaptation of the pancreatic islets to pregnancy results in enhanced insulin secretion capacity. It begins early in pregnancy, prior to the development of insulin resistance, and mediates the process of facilitated anabolism (1, 2). Later in pregnancy, physiological insulin resistance necessitates a compensatory increase in insulin secretion to maintain normoglycemia (1, 3–5). Failure of islet beta-cell adaptation results in gestational diabetes which affects 6% to 15% of all pregnancies globally (6). Thus, better understanding of the pathophysiology underlying gestational diabetes development first requires comprehensive knowledge of the mechanisms by which pancreatic islets, in particular beta-cells, adapt to normal pregnancy.

Significant progress has been made in understanding the mechanisms of islet adaptation to pregnancy (7, 8). Increased insulin secretion is supported by both islet beta-cell expansion as well as enhanced function, including lowering of the glucose set-point for insulin secretion (7–11). Mouse islet gene transcription studies have been invaluable in elucidating mechanisms (12, 13). It is now clear that in rodents, enhanced prolactin receptor signaling, intracellular serotonin synthesis and signaling, as well as dependence on glucagon-like peptide 1 receptor, are important for successful islet adaptation to pregnancy (9, 14–17). Estrogen may also have a role in protecting the increased beta-cell mass of pregnancy (18, 19). However, the involvement of these particular signaling pathways in islet adaptation in human pregnancy is less clear, such that alternate or additional factors may be involved (20). The islet beta-cell has substantial capacity to sense multiple metabolic and hormonal inputs, such that other humoral factors may be involved in adaptation processes (20, 21). Relevant to human pregnancy, serum from pregnant women from the later stages of pregnancy has been shown to readily activate cell proliferation of rat beta-cells (22). Serum lipid moieties, which are markedly elevated in pregnancy, could be candidate factors for promoting islet adaptation (23, 24).

In support of a role for lipids in beta-cell adaptation to pregnancy, previous studies have shown that neonatal rat islets exposed to prolactin are highly responsive to fatty acids with respect to beta-cell proliferation and enhanced beta-cell function (25, 26). Additionally, it is well established that exogenous fatty acids augment glucose-stimulated insulin secretion (GSIS) in non-pregnant rodent islets *via* activating free fatty acid receptors such as Ffar1 (also known as Gpr40) and through generation of intracellular lipid signaling molecules for secretion (27, 28). Furthermore, islet beta-cell compensation to severe insulin resistance in the non-pregnant, genetic obese, Zucker fatty (ZF) rat model is associated with enhanced glucose flux through the anaplerotic pathway (29) and enhanced lipid signalling generated by glycerolipid/fatty acid (GL/FA) cycling (30).

As the role of islet GL/FA cycling and lipid signalling in islet beta-cell adaptation in normal pregnancy has not been investigated, the present study focused on the effects of fatty acids on GSIS and on the major pathways of intracellular lipid partitioning in islets of early pregnant (gestational day 11, G11) and late pregnant (gestational day 19, G19) Sprague-Dawley rats. The expression of key beta-cell genes including transcription factors and enzymes involved in beta-cell glucose and lipid metabolism were also evaluated.

## Materials and Methods

### Animals

Female age-matched time-mated pregnant (11-12 weeks of age at day 0 of pregnancy) and virgin Sprague-Dawley rats were either purchased from the Monash University Animal Facility (Clayton, Vic., Australia) and housed in the animal facility at the Royal Melbourne Hospital (Parkville, Vic, Australia) (total number used, 13 virgin and 14 pregnant), or purchased from the Animal Resource Centre (Perth, WA, Australia) and housed in the Canberra Hospital Animal Facility (Garran, ACT, Australia) [total number used, 80 virgin, 65 pregnant (G11), 74 pregnant (G19)]. All rats were accommodated in a temperature-controlled environment (22°C) and were subject to controlled lighting (12h dark/12 h light). Animals had free access to water and standard laboratory chow (Barastoc, Pakenham, Vic., Australia for the Parkville studies and Gordon’s Specialty Stockfeeds, NSW, Australia for the Garran studies). All animal procedures were performed in compliance with the Australian code of practice for care and use of laboratory animals for scientific purposes. The project was approved by the Royal Melbourne Hospital Animal Ethics Committee and the Australian National University Animal Experimental Ethics Committee (Project F.MS.17.08).

### Oral Glucose Tolerance Tests and Prolonged Fasting Studies in Chronically Catheterized Conscious Rats

Oral glucose tolerance tests (OGTT) and prolonged fasting studies were performed in rats housed at the Royal Melbourne Hospital Animal Facility. Pregnant rats at gestational day 12 and age-matched virgin rats were anesthetized with 60 mg/kg intraperitoneal pentobarbitone (Boehringer Ingelheim, Artarmon, NSW, Australia), and a right jugular catheter into the right atrium was inserted for blood sampling, as previously described (31). The catheters were flushed with 0.9% NaCl containing 10U/ml heparin (Bull Laboratories, Mulgrave, Vic, Australia), exteriorized towards the back of the neck, and then sealed for later use. The catheterized rats were housed in specially designed metabolic cages and allowed to recover (31). The rats were trained to drink 1.4 ml of 50% glucose (containing 0.7 g glucose) at 2, 4 and 5 days post catheter insertion.

Oral glucose tolerance tests (OGTTs) and prolonged fasting studies were performed on day 7 following catheter insertion at which time the pregnant rats were at day 19 of gestation (G19). Rats were weighed and laboratory chow diet was removed at 0600 h, the sampling jugular catheter was connected *via* tube extension to a peristaltic pump (Gilson Minipuls 2, Villiers, Lebvel, France) at 0800 h, and the experiments commenced at 1000 h (time -120 min). The sampling catheter and connecting tubing were filled with heparinized saline to maintain patency between blood samples. To avoid anemia and intravascular volume depletion, the red cells from all blood samples were resuspended in heparinized saline and transfused back to the same rat periodically through the OGTT procedure. The rats were allowed to move freely within the confines of the metabolic cages throughout the experiments without need for handling from time -120 min.

At time 0 min (after 6 h of fasting) the rats were presented with a dish containing 1.4 ml of 50% glucose (OGTT), or 1.4 water (prolonged fasting), which they drank within 1 to 2 min, or 5 min, respectively. For the OGTT, blood samples (up to 300 µl) were taken 10 minutely from time -20 to 0 min, 5 minutely from time 0 to 20 min, 10 minutely from time 20-60 min, and 15 minutely from time 60 to 120 min. Plasma glucose was measured in all samples in the OGTT studies and the -20, 0, 30, 60, 90, and 120 min samples in the prolonged fasting studies. Plasma insulin was measured in the -20, 0, 15, 30, 60, 90 and 120 min samples in the OGTT studies, and in the 0, 60 and 120 min samples in the prolonged fasting studies.

Plasma glucose was determined using a 23 AM YSI Glucose Analyser (Yellow Springs Instrument Company, Yellow Springs, Ohio, USA). Plasma insulin by radioimmunoassay using a first and second antibody method (Phadeseph Pharmacia, Uppsala, Sweden).

### Liver Glycogen Content

On completion of the OGTT and prolonged fasting studies (1400 h), rats were euthanized with an overdose of pentobarbitone given intravenously and the livers were quickly excised, weighed, frozen in liquid nitrogen and stored at -80^0^C. Glycogen content was measured using a modification of the method of Keppler and Decker, as previously described (32).

### Fed State Blood Biochemistry

Fed state blood biochemistry and islet isolation experiments were performed in rats housed at the Canberra Hospital Animal Facility. Between 0830 h and 0930 h, prior to anesthesia for islet isolation, fed virgin and pregnant rats (G11 and G19) were weighed and a 300 µl tail vein blood sample was taken for biochemistry analyses. Blood glucose was measured using a glucose meter (ACCU-CHEK Advantage II^®^; Roche, Mannheim, Germany) at the time of sampling. Plasma insulin was measured by radioimmunoassay ([^125^I]-insulin tracer from Millipore (MA, USA), guinea pig anti-rat insulin serum and guinea pig serum from Jackson ImmmunoResearch Laboratories Inc. (PA, USA), and goat anti-guinea pig IgG from Equitech-Bio Inc. (TX, USA). Commercial enzymatic colorimetric assays were used to measure serum non-esterified fatty acids (NEFA) (NEFA Kit; Wako Chemicals, Osaka, Japan) and serum triglycerides (GPO Trinder; Sigma-Aldrich, Saint Louis, MS, USA).

### Pancreatic Islet Isolation

After fed state blood sample collection, rats were anesthetized using a combination of ketamine (Troy Laboratories, NSW, Australia) at 100 mg/kg body weight and xylazine at 20 mg/kg of body weight (Xylazil, Troy Laboratories, NSW, Australia) and euthanized by exsanguination. Islets were handpicked using a stereomicroscope after collagenase digestion and histopaque gradient separation (33). Prior to experiments, islets were cultured for 1 h in RPMI-1640 medium supplemented with 10% fetal calf serum, 10 mmol/l HEPES (pH 7.4), 1 mmol/l sodium pyruvate, 100 U/ml penicillin and 100 µg/ml streptomycin (RPMI complete medium) with 11 mmol/l glucose at 37°C in a humidified atmosphere containing 5% CO_2_.

### Preparation of BSA-Bound Palmitate

Sixty seven mg of the sodium salt of palmitic acid (Nu-Chek Prep, Inc., MN, USA) was dissolved in 10 mL Krebs Ringer Bicarbonate buffer with 10 mmol/L HEPES at pH 7.4 (KRBH) with 5% fatty acid-free bovine serum albumin BSA (weight:volume; Sigma Aldrich, CA, USA) and incubated under N_2_ gas at 37°C room for 16 hours as previously described (34). The BSA-bound fatty acid solution was filtered through a 0.22 μm filter after which the palmitate concentration was measured using a NEFA C Kit (Wako chemicals, Osaka, Japan). The stock solution was diluted down to a final concentration of 4 mmol/l using KRBH with 5% fatty acid-free BSA and aliquoted into microcentrifuge tubes, gassed briefly under a stream of N_2_, sealed and stored at -20°C until use.

### Insulin Secretion and Islet Insulin Content in Isolated Islets

Freshly isolated islets (6/well in a 24-well plate, three technical replicates per data point) were used to measure static insulin secretion at 3, 8 and 16 mmol/l glucose (Baxter, IL, USA) in the presence and absence of 0.25 mmol/l BSA-bound palmitate, as previously described (30). The islets were rested in RPMI complete medium with 11 mmol/l glucose for 1 h followed by 3 mmol/l for 2 h at 37°C with 5% CO_2_. The islets were then washed in KRBH supplemented with 0.5% fatty acid-free BSA and 3 mmol/l glucose and pre-incubated in 1 ml of the same media for 40 min at 37°C with 5% CO_2_. After further washing, islets were submitted to 45 min incubation in 1 ml/well KRBH buffer supplemented with 3, 8 and 16 mmol/l glucose and 0.25% fatty acid-free BSA, in the presence and absence of 0.25 mmol/l BSA-bound palmitate. At the end of the incubation period, supernatant was collected from all wells for quantitation of secreted insulin. Islet intracellular insulin content was extracted by acidified ethanol (0.2 mmol/l HCl in 75% ethanol). Secreted insulin and islet insulin contents were quantified by radioimmunoassay.

### Measurement of Palmitate Oxidation in Isolated Islets

Islet fatty acid oxidation was determined by measuring the release of ^3^H_2_O from [9, 10(n)-^3^H] palmitate tracer (PerkinElmer, MA, USA), as previously described (30). Freshly isolated islets (50/well in a 12-well plate, three technical replicates per data point) were rested for 1 h in RPMI complete with 11 mmol/l glucose at 37°C with 5% CO_2_. Islets were then transferred to fresh 12-well plates containing 1 mL of RPMI complete with 5.5 mmol/L glucose, 0.25% fatty acid-free BSA, 0.1 mmol/L BSA-bound palmitate and 2 µCi/ml [9, 10(n)-^3^H] palmitate tracer (PerkinElmer, MA, USA) and incubated for 16 h at 37°C with 5% CO_2_ to achieve similar intra- and extracellular fatty acid specific activities. The islets were then washed in KRBH supplemented with 0.25% BSA, 0.1 mmol/l BSA-bound palmitate and 3 mmol/l glucose after which they were incubated for 2 h in 1 ml/well of oxidation media containing KRBH supplemented with 0.25% BSA, 0.1 mmol/l BSA-bound palmitate, 1 mmol/l L-carnitine hydrochloride (Sigma Aldrich, CA, USA), and 2 µCi/ml [9,10(n)-^3^H] palmitate tracer at 3, 8 or 16 mmol/l glucose at 37°C with 5% CO_2_. An additional 300 µl of oxidation media was set aside for quantitation of the oxidation media palmitate specific activity. Media from all wells, centrifuged to remove any islet debris, were then collected for later quantitation of ^3^H_2_O. The islets were washed three times with phosphate buffered saline (PBS). Islet pellets were lysed by adding 50 µl of protein lysis buffer and then stored at -20°C for subsequent determination of islet protein content.

Quantitation of ^3^H_2_O in the oxidation experiment media involved transferring 800 µl of the media into capless 1.5 ml Eppendorf tubes which were then acidified with 80 µl concentrated HCl (Sigma Aldrich, CA, USA), as previously described (30). The uncapped Eppendorf tubes were then carefully placed inside 20 ml scintillation vials (PerkinElmer, MA, USA) containing 500 µl H_2_O. Vials were capped then incubated at 50°C with agitation for 24 h to allow equilibration of ^3^H_2_O between the media in the tube and the H_2_O in the vial. Eppendorf tubes containing known amounts of ^3^H_2_O (PerkinElmer, MA, USA) were used to estimate % recovery by this method which was usually about 28%. Following incubation, vials were cooled for 1 h to room temperature and the Eppendorf tubes were carefully removed and discarded. 5 ml scintillation fluid (Beckman Coulter, CA, USA) was added to each vial and the ^3^H_2_O cpm was measured in a β-scintillation counter (Packard, ACT, Australia). Scintillation counting of 100 μl of oxidation media (in triplicate) was also performed to determine palmitate specific activity. The rate of palmitate oxidation (nmol/h) was calculated as the ^3^H_2_O produced (cpm/h) divided by the specific activity of the palmitate in the oxidation media (cpm/nmol).

### Measurement of Palmitate Esterification in Isolated Islets

Islet fatty acid esterification was determined by measuring the incorporation of [1-^14^C] palmitate tracer (PerkinElmer, MA, USA) into glycerolipids, as previously described (30). Freshly isolated islets (50/well in a 12-well plate, three technical replicates per data point) were rested for 1 h in RPMI complete with 11 mmol/l glucose at 37°C with 5% CO_2_. The islets were then washed with 1 ml/well RPMI complete with 3 mmol/l glucose and incubated for 16 h in esterification media containing 1 ml RPMI complete with 3, 8 or 16 mmol/l glucose, 0.25% fatty acid-free BSA, 0.1 mmol/l BSA-bound palmitate and 1 µCi/ml [1-^14^C] palmitate tracer at 37°C with 5% CO_2._ An additional 300 µl of esterification media was set aside for quantitation of palmitate specific activity. Following the 16 h incubation, islets were collected in minimal volume and washed in cold PBS containing 0.1% fatty acid-free BSA.

The pelleted islets were resuspended in 3 ml Folch reagent, as previously described (30). Total lipids were extracted and non-polar lipids were separated by thin-layer chromatography. Incorporation of labelled palmitate into phospholipids (PL), cholesterol esters (CE), monoacylglycerols (MAG), diacylglycerols (DAG) and triacylglycerols (TG) was quantified after scraping of bands from the plates and ß-scintillation counting. Net esterification rates (nmol) were calculated as the incorporated [1-^14^C] palmitate (cpm) divided by the specific activity of the palmitate in the esterification media (cpm/nmol).

### Measurement of Lipolysis in Isolated Islets

Islet lipolysis was determined by measuring the rate of glycerol release from islets, as previously described (35). Freshly isolated islets (60 islets/well in a 12-well plate, three technical replicates per data point) were rested in RPMI complete with 11 mmol/l glucose for 1 h and 3 mmol/l for 2 h at 37°C with 5% CO_2_. Islets were then washed in KRBH containing 0.5% fatty acid-free BSA at 3 mmol/l glucose and pre-incubated for 40 min in 1 ml KRBH containing 0.5% fatty acid-free BSA at 3 mmol/l glucose, washed again and then transferred to 96-well plates to a final volume of 300 µl KRBH containing 0.5% fatty acid-free BSA with 3 or 16 mmol/l glucose in the presence or absence of 0.25 mmol/l BSA-bound palmitate. Plates were incubated for 3 hours at 37°C in a humidified atmosphere with 5% CO_2_, after which 150 µl samples of media were collected for glycerol determination (35). For background measurements, 100 µl of media were collected from all wells at 5 min into the incubation. Islets were collected and rinsed twice in PBS containing 0.1% fatty acid-free BSA, then resuspended in 50 µl of protein lysis buffer for protein determination.

### Measurement of Islet Protein and Triacylglycerols Content

Batches of 15 freshly isolated islets from G11 and G19 pregnant, and virgin rats (single sample per rat) were used for determination of islet protein content using the Pierce BCA Protein Assay Kit (Thermo Scientific, IL, USA). Batches of 100 freshly isolated islets (single sample per rat) were used for determination of islet total TG using the Serum TG Kit (Sigma Aldrich, CA, USA), as previously described (30).

### Islet RNA Analysis

Total islet RNA was extracted using the RNeasy Micro Kit (Qiagen, Ontario, Canada) with on-column DNase digestion (Qiagen) according to the manufacturer’s instructions. RNA integrity was assessed on a denaturing agarose gel with ethidium bromide staining. RNA (1-3µg) was reverse transcribed to cDNA with random primers and reverse transcription mix according to manufacturer’s instructions (Invitrogen, CA, USA). Primers were designed using Primer Express Software (Applied Biosystems, CA, USA) and Primer-BLAST (http://www.ncbi.nlm.nih.gov/tools/primer-blast/) and ordered from GeneWorks (VIC, Australia) (**Supplementary Table 1**). Realtime PCR was performed in a 384-well plate on the 7900 HT Real Time PCR System (Applied Biosystems, CA, USA) using standard reaction cycle conditions. The 8 μl reaction volume containing 3 μl of master mix containing 0.25 μl of forward primer, 0.25 μl of reverse primer, 2.5 μl of 2x Power SYBR Green Kit (Applied Biosystems, CA, USA) and 2 μl of diluted cDNA was prepared using automated pipetting on the epMotion 5070 (Eppendorf, Hamburg, Germany). The value obtained for each specific gene product (single sample per rat) was expressed relative to the house keeping gene β-actin.

### Statistical Analysis

All values are presented as means ± SEM. For islet studies, the mean of technical replicates was used for each data point. Prior to analysis, all continuous variable data were tested for normal distribution and transformed to account for skewed distribution if required. Statistical analyses were performed using unpaired student t-test, one-way analysis of variance (ANOVA), two-way ANOVA including with repeated measures for time course analyses, and Bonferroni *post-hoc* testing, using Prism software version 9 (GraphPad Software, San Diego, CA, USA). Two-way and Three-way ANOVA analyses of islet studies, with Bonferroni *post-hoc* testing for multiple comparisons between rat groups, were performed using IBM SPSS Statistics version 25.0 (IBM Corporation, Armonk, NY, USA). A *P* value of <0.05 was considered significant.

## Results

### Body Weight and Fed-State Blood Biochemistry: Late Rat Pregnancy Is Characterized by Hypoglycemia, Hyperinsulinemia and Hypertriglyceridemia

**Figure 1:**
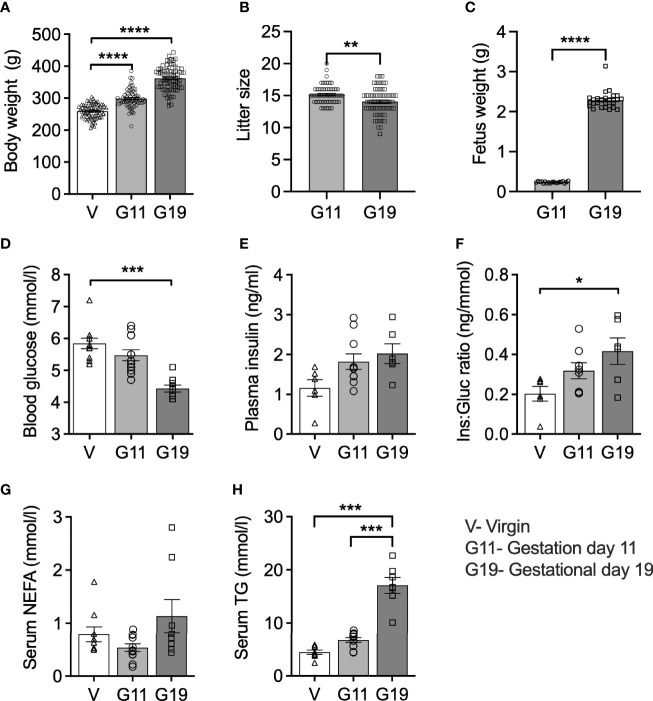
Body weight and 9AM fed state blood biochemistry in virgin (V), gestational age day 11 (G11) and gestational age day 19 (G19) rats. Body weight **(A)**, litter size **(B)**, (n=65-80 per group). Mean litter fetus weight **(C)** (n=19-27 per group). Blood glucose **(D)**, plasma insulin **(E)**, plasma insulin:blood glucose ratio (Ins : Gluc ratio) **(F)**, (n=6-12 per group). Serum non-esterified fatty acids (NEFA) **(G)**, serum triglycerides (TG) **(H)**, (n=7-11 per group). Means ± SEM; one-way ANOVA with Bonferroni *post-hoc* testing **(A, D–H)**; unpaired t-test **(B, C)**; *P<0.05, **P<0.01, ***P<0.001; ****P<0.0001.

Maternal body weight increased in pregnant rats by 15% at G11 and by 39% at G19 compared to age-matched virgin rats (**Figure 1A**). Litter size reduced minimally from a mean of 15 to 14 fetuses from G11 to G19, suggesting minimal fetus resorption rates (**Figure 1B**). Fetus weight increased by 10-fold from G11 to G19 (**Figure 1C**), such that the combined weight of fetuses per litter, expressed as a percentage of maternal body weight, increased on average from 1.2% at G11 to 8.8% at G19. Fed (0900 h) blood glucose concentrations fell progressively throughout pregnancy being 26% lower in G19 (4.4 ± 0.1 mmol/l) compared to virgin rats (5.8 ± 0.2 mmol/l) (**Figure 1D**). Fed plasma insulin levels trended higher in pregnancy being 57% and 74% higher at G11 and G19, respectively (1.82 ± 0.20 and 2.02 ± 0.25 ng/mL), compared to virgin rats (1.2 ± 0.2 ng/ml) (G11 vs virgin, *P*=0.13; G19 vs virgin *P*=0.06) (**Figure 1E**). The ratio of non-fasting plasma insulin to blood glucose concentration increased by 110% at G19, compared to virgin rats (G19 vs virgin *P*<0.05) (**Figure 1F**). There was a non-significant trend for decreased non-fasting serum NEFA at G11 of pregnancy compared to virgin and G19 rats (**Figure 1G**). Non-fasting serum TG concentration progressively increased during pregnancy being 51% and 284% higher at G11 and G19, respectively (6.7 ± 0.5 and 17.1 ± 1.5 mmol/l), compared to virgin rats (4.4 ± 0.4 mmol/l) (G11 vs virgin, *P*=0.14; G11 vs G19, *P*=0.13; G19 vs virgin, *P*<0.0001) (**Figure 1H**).

### Plasma Glucose Is Lower During Oral Glucose Tolerance Testing in Late Rat Pregnancy

**Figure 2:**
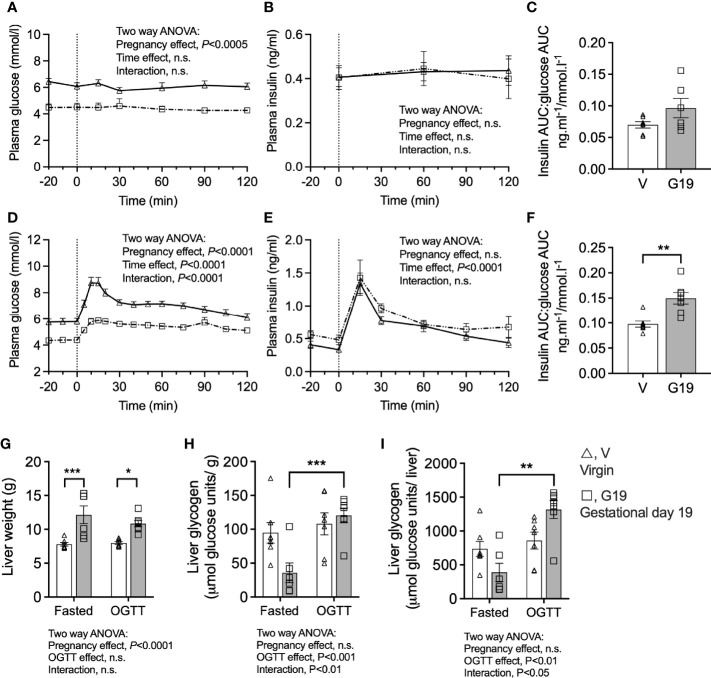
Prolonged fasting and oral glucose tolerance tests in chronically catheterized conscious virgin (V), and gestational age day 19 (G19) rats. Plasma glucose **(A)**, plasma insulin **(B)**, plasma insulin area under the curve (AUC):plasma glucose AUC ratio **(C)**, in rats fasted for 8 h at time 120 min, (n=6-7 per group). Plasma glucose **(D)**, plasma insulin **(E)**, plasma insulin area under the curve (AUC):plasma glucose AUC ratio **(F),** in rats fasted for6 h and given oral glucose (0.7 g) at time 0 min, (n=7 per group). Means ± SEM; repeated measure two-way ANOVA **(A, B, D, E)**; unpaired t-test **(C, E)**; two-way ANOVA with Bonferroni *post-hoc* testing **(G–I)**; n.s., not significant, *P<0.05, **P<0.01, ***P<0.001.

Plasma glucose concentrations (1200 h) of the 6 h fasted rats (time 0 min) were 24% lower in G19 pregnant (4.4 ± 0.2 mmol/l) compared to virgin (5.8 ± 0.3 mmol/l) rats (**Figure 2A**). Fasting plasma insulin concentrations were not different between the G19 and virgin rats (**Figure 2B**). Fasting for another 2 h (total 8 h of fasting) was not associated with changes in either plasma glucose or insulin concentrations in pregnant or virgin rats (**Figures 2A, B**). The ratio of fasting plasma insulin to plasma glucose trended 38% higher in the G19 pregnant compared to virgin rats, but this was not significant (*P*=0.11) (**Figure 2C**). After 0.7g of oral glucose, glycemia remained lower in the G19 pregnant compared to virgin rats (area under the curve 0-120 min (AUC_0-120_); 655 ± 24 versus 849 ± 13 mmol.l^-1^.min; *P*<0.0001) (**Figure 2D**), however, despite the lower glycaemia, the insulin response to the oral glucose of G19 pregnant compared to virgin rats did not differ (AUC_0-120_; 97 ± 7 compared to 83 ± 7 ng.ml^-1^.min; not significant) (**Figure 2E**). Consistent with islet beta-cell adaptation to pregnancy, the ratio of the AUC_0-120_ of plasma insulin to the AUC_0-120_ plasma glucose was 53% higher in the G19 compared to virgin rats (**Figure 2F**).

### Liver Glycogen Content: Evidence of Accelerated Starvation and Facilitated Anabolism in Late Pregnant Rats

The livers of G19 pregnant compared to virgin rats were 46% heavier (**Figure 2G**). The livers of fasted G19 pregnant rats were deplete of glycogen, whether expressed per g liver (**Figure 2H**) or per liver (**Figure 2I**). Liver glycogen content 2 h after the 0.7 g oral glucose load recovered in the pregnant rats (**Figures 2H, I**). Expressed per whole liver, from the fasted to the post-glucose load state, liver glycogen increased by 139% in the G19 pregnant rat livers (*P*<0.001) compared to only 17% in the virgin rat livers (not significant) (**Figure 2I**).

### Pancreatic Islet Characteristics: Larger Islets With Reduced Triacylglycerols Content in Late Pregnancy

**Figure 3:**
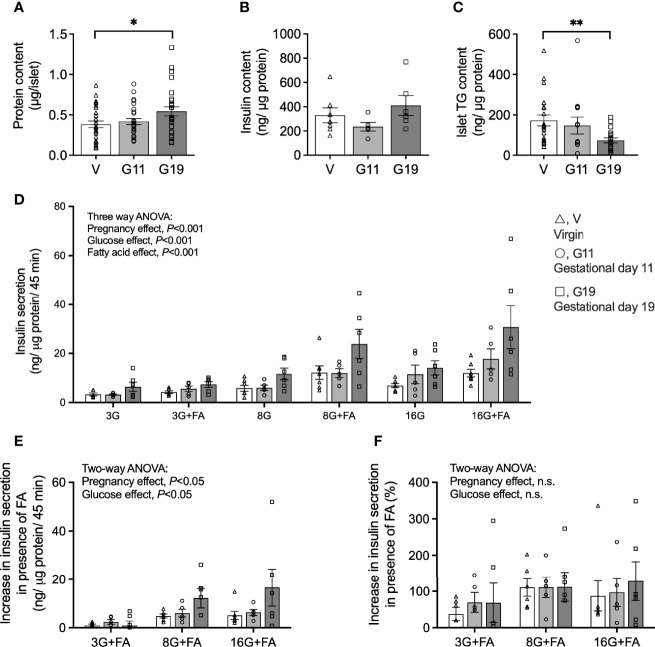
Insulin secretion in isolated pancreatic islets from virgin (V), gestational age day 11 (G11) and gestational age day 19 (G19) rats. Islet protein content **(A)** (n=26-28 rats per group), insulin content **(B)** (n=5-7 per group), triacylglycerols content **(C)** (n=13-22 rats per group). Static insulin secretion at glucose concentrations of 3 (3G), 8 (8G) and 16 (16G) mmol/l in the absence or presence of 0.25 mmol/l BSA-bound palmitate (+FA) **(D)**, (n=5-7 rats per group). Absolute increase in insulin secretion **(E)**, and percentage increase in insulin secretion **(F)**, in the condition in the presence of palmitate (3G+FA, 8G+FA, 16G+FA) over the respective condition in the absence of palmitate (3G, 8G, 16G), (n=5-7). Means ± SEM; one-way ANOVA with Bonferroni *post-hoc* testing **(A–C)**; three-way ANOVA; multiple comparisons; G11 vs virgin not significant, G19 vs virgin *P*<0.001, G19 vs G11 *P*<0.01 **(D)**; and two-way ANOVA **(E, F)**; n.s., not significant, *P<0.05, **P<0.01.

Islet total protein content was unaltered in G11 rats compared to age-matched virgin rats (0.42 ± 0.04 vs 0.38 ± 0.04 µg protein/islet, respectively), but was significantly increased by 42% in G19 rats (0.54 ± 0.01 µg protein/islet) (G19 vs virgin, *P*<0.05) (**Figure 3A**). The islet total insulin content, after normalization by islet protein content, trended lower in islets of G11 pregnant rats and higher in G19 pregnant rat, respectively (235 ± 35 and 410 ± 82 ng/µg protein, respectively) compared to the virgin islets (329 ± 62 ng/µg protein), although the changes were not significant (one-way ANOVA, *P*=0.17) (**Figure 3B**). Islet TG content from fed rats, normalized by protein content, was similar in G11 pregnant compared to virgin rats, respectively (147 ± 42 and 172 ± 27 ng/µg protein), but was reduced by 51% in G19 rats (73 ± 13 ng/µg protein) (G19 vs virgin, *P*<0.01) (**Figure 3C**).

### Fatty Acids Augment Glucose-Stimulated Insulin Secretion in Isolated Islets of Pregnant Rats

Glucose-stimulated insulin secretion (GSIS) expressed per islet protein content is shown in **Figure 3D** and per islet and total islet insulin content in Supplemental **Figures 1A, B**. GSIS expressed per islet protein shows firstly that overall insulin secretion was increased by higher glucose concentrations and that this was augmented in the presence of palmitate in the incubation media (three-way ANOVA: glucose and fatty acid main effects, *P*<0.001 for both) (**Figure 3D**). Importantly, there was also an effect of pregnancy to increase GSIS (three-way ANOVA: pregnancy main effect, *P*<0.001), and this effect was driven by islets of G19 rather than G11 pregnant rats (multiple comparisons; G11 vs virgin, not significant; G19 vs virgin, *P*<0.001; G19 vs G11, *P*<0.01) (**Figure 3D**). GSIS from isolated islets of G19 rats were approximately 2-fold higher than that of virgin rats at both 3 and 8 mmol/l glucose concentrations and 2.5-fold higher at 16 mmol/l glucose concentration (**Figure 3D**). GSIS normalized per islet was 3-fold higher at 16 mmol/l in the absence of fatty acid and 4-fold higher at 16 mmol/l in the presence of fatty acid in late pregnant compared to virgin rat islets (**Supplementary Figure 1A**). GSIS in islets of mid-pregnancy rats, expressed by either islet protein or per islet, did not appear to be altered from GSIS measured in virgin rat islets. Expressed per islet total insulin content, however, effects of earlier pregnancy on GSIS were evident (multiple comparisons; G11 vs virgin, *P*<0.005; G19 vs virgin, not significant; G19 vs G11, *P*<0.05) (**Supplementary Figure 1B**). This was likely the result of the trend for lower insulin content corrected for islet protein content in the G11 compared to G19 rat islets (42% lower, *P=*0.19). The insulin content of G11 rat islets was not different to that of virgin rat islets (**Figure 3B**).

To look further into whether pregnancy modulates the effect of fatty acids to augment GSIS, we quantified the increase in GSIS, expressed per islet protein content, for each glucose concentration in the presence compared to the absence of palmitate. Quantified as an absolute GSIS increase in the presence of palmitate for the respective glucose concentrations, a pregnancy effect was evident (two-way ANOVA: pregnancy main effect, *P*<0.05) (**Figure 3E**), but a pregnancy effect was not evident if the FA augmentation of GSIS was quantified as a percentage (**Figure 3F**).

### Net Palmitate Esterification Into Phospholipids, Monacylglycerols and Triacylglycerols Is Increased Is Islets of Pregnant Rats

**Figure 4:**
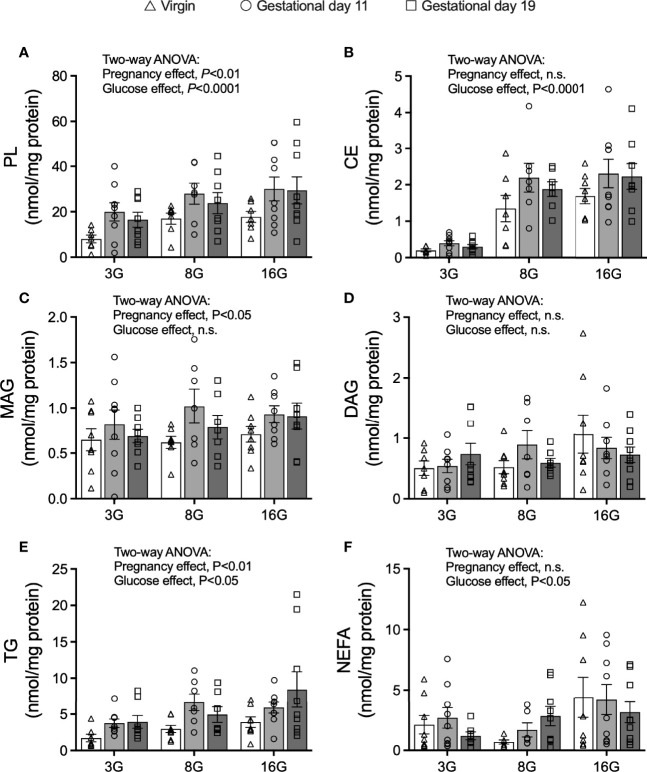
Fatty acid esterification into glycerolipids in isolated pancreatic islets from virgin (V), gestational age day 11 (G11) and gestational age day 19 (G19) rats. Net fatty acid esterification into phospholipids (PL) **(A)**, cholesterol esters (CE) **(B)**, monoacylglycerides (MAG) **(C)**, diacylglycerides (DAG) **(D)**, triacylglycerols **(E)**, and islet non-esterified fatty acid content **(F)** in islets incubated with [1-^14^C] palmitate esterification mix (as described in materials and methods) at glucose concentrations of 3 (3G), 8 (8G) and 16 (16G) mmol/l for 16 h, (n=7-9 rats per group). Means ± SEM; Two-way ANOVA; multiple comparisons; G11 vs virgin *P*<0.01, G19 vs virgin *P*<0.05, G11 vs G19 not significant **(A)**; G11 vs virgin *P*<0.05, G19 vs virgin not significant, G11 vs G19 not significant **(C)**; G11 vs virgin *P*<0.05, G19 vs virgin *P*<0.02, G11 vs G19 not significant **(E)**; n.s., not significant.

As palmitate esterification was measured over 16 h, the results reflect net accumulation of labelled palmitate from the incubation media considering that esterification and lipolysis occur together (30). Net palmitate esterification into phospholipids (PL), cholesterol esters (CE) and TG were increased by elevated glucose concentrations (Two-way ANOVA: glucose main effect, *P*<0.0001 for PL and CE, P<0.05 for TG) (**Figures 4A, B, E**). No significant changes were seen in the levels of monoacylglycerols (MAG) and diacylglycerols (DAG) in response to elevated glucose concentrations (**Figures 4C, D**). Importantly, there was an effect of pregnancy on net palmitate esterification into PL, MAG and TG (Two-way ANOVA: pregnancy effect, *P*<0.01 for PL and TG, significant in *post-hoc* testing for both G11 and G19 compared to virgin rat islets; *P*<0.05 for MAG, significant for G11 compared to virgin rat islets only) suggesting their potential importance as lipid signaling processes in fatty acid augmented GSIS in pregnancy (**Figures 4A, C, E**). Palmitate esterification into TG in G19 pregnant rat islets was double that of virgin rat islets when assessed at 16 mmol/l glucose (8.4 ± 2.5 vs 3.9 ± 0.9 nmol/mg protein, respectively; *P<*0.02 *post-hoc* testing) (**Figure 4E**). Elevated glucose concentrations were associated with an increase in islet non-esterified labelled palmitate content [an index of cellular long chain-CoA (30)] (Two-way ANOVA: glucose main effect, *P*<0.05), however, there was no evidence of a pregnancy effect on this measure (**Figure 4F**).

### Fatty Acid Oxidation Is Reduced in Islets of Pregnant Rats Whereas Effects on Lipolysis Vary According to Gestational Age

**Figure 5:**
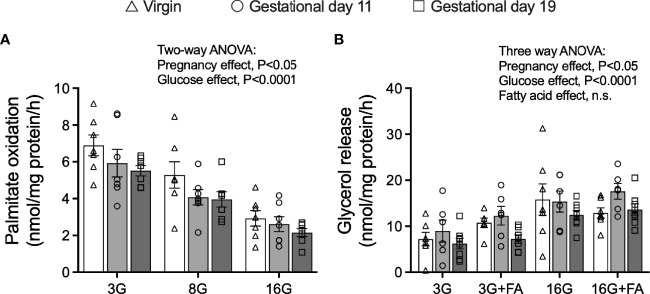
Fatty acid oxidation and rates of lipolysis in isolated pancreatic islets from virgin (V), gestational age day 11 (G11) and gestational age day 19 (G19) rats. Islets were pre-labelled for 16 h with [9,10(n)-^3^H] palmitate and fatty acid oxidation was then measured from a 2 h incubation in [9,10(n)-^3^H] palmitate oxidation mix (as described in materials and methods) at glucose concentrations of 3 (3G), 8 (8G) and 16 (16G) mmol/l **(A)**, (n=7 rats per group). Lipolysis was measured from the rate of glycerol release over 3 h in islets incubated at glucose concentrations of 3 (3G), 8 (8G) and 16 (16G) mmol/l in the absence or presence of 0.25 mmol/l BSA-bound palmitate (+FA) **(B)**, (n= rats 6-8 per group). Means ± SEM; two-way ANOVA; multiple comparisons; G11 vs virgin not significant, G19 vs virgin *P*<0.02, G19 vs G11 not significant **(A)**; three-way ANOVA; multiple comparisons; G11 vs virgin not significant, G19 vs virgin not significant, G19 vs G11 *P*<0.02 **(B)**; n.s., not significant.

Islet palmitate oxidation was measured after pre-labelling the islets with [9, 10(n)-^3^H] palmitate labelled exogenous cold palmitate for 16 h as previously described (30). The 16 mmol/l compared to 3 mmol/l glucose concentration suppressed fatty acid oxidation by approximately 60% in all groups (Two-way ANOVA: glucose main effect, *P*<0.0001) (**Figure 5A**). Moreover, palmitate oxidation, assessed over all glucose concentrations, was lower in islets from pregnant rats compared to virgin rats (Two-way ANOVA: pregnancy main effect, *P*<0.05, significant in *post-hoc* testing for G19 compared to virgin rats only) (**Figure 5A**). In all groups, the rate of lipolysis, as measured by islet glycerol release) was higher in islets incubated at 16 mmol/l compared to 3 mmol/l glucose, with higher rates being evident in islets from G11 compared to G19 pregnant rats (three-way ANOVA: glucose main effect *P*<0.0001, pregnancy main effect *P*<0.05, multiple comparisons, G11 compared to G19, *P*<0.02, **Figure 5B**).

### Expression of Key Islet Genes at the mRNA Level: *Ppargc1a* Is Reduced in Rat Pregnancy

**Figure 6:**
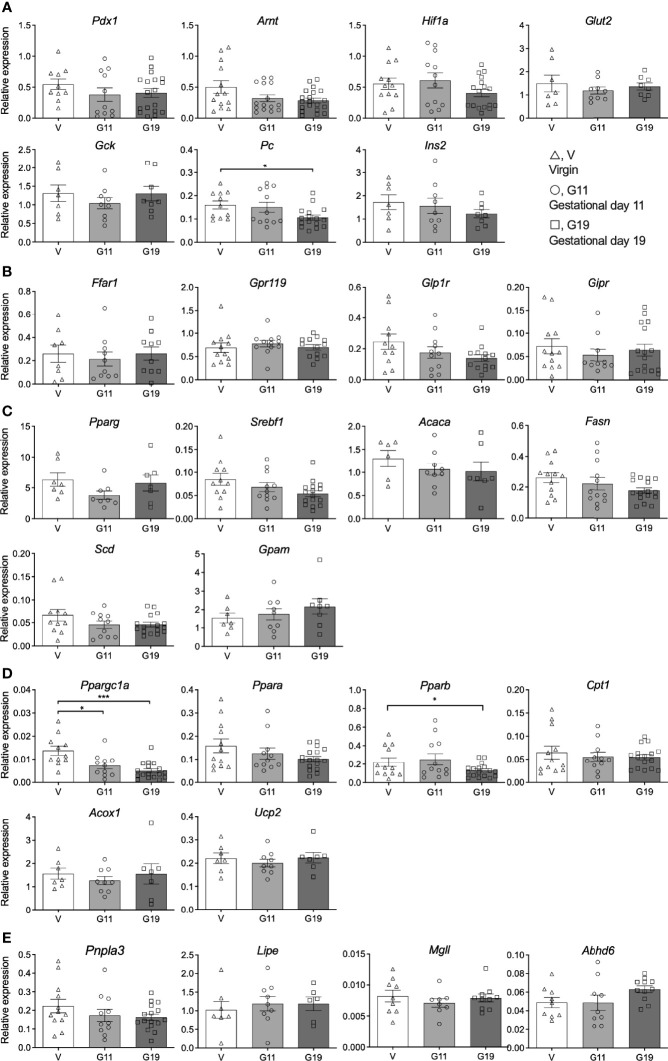
Islet gene expression in isolated pancreatic islets from virgin (V), gestational age day 11 (G11) and gestational day 19 (G19) rats. Islet beta-cell transcription and function genes **(A)**, fatty acid receptor and incretin receptor genes **(B)**, fatty acid synthesis and esterification genes **(C)**, fatty acid oxidation genes **(D)**, lipolysis genes **(E)**, (n= 6-18 rats per group). Results expressed relative to the house keeping gene β-actin. Means ± SEM; one-way ANOVA with Bonferroni *post-hoc* testing; *P<0.05, ***P<0.001.

Expression of islet beta-cell transcription and function genes (**Figure 6A**), fatty acid receptor and incretin receptor genes (**Figure 6B**), fatty acid synthesis and esterification genes (**Figure 6C**), fatty acid oxidation genes (**Figure 6D**) and lipolysis genes (**Figure 6E**) was assessed in isolated islets of the virgin, G11 and G19 rats. The most striking result was an effect of pregnancy to reduce the expression of *Ppargc1a* which encodes the protein peroxisome proliferator-activated receptor gamma coactivator 1-alpha (**Figure 6D**). Expression of *Ppargc1a* was reduced by 51% in G11 and 64% in G19 pregnant rat islets compared to virgin rat islets (**Figure 6D**). This was associated with a concomitant 38% reduction in *Pparb* expression in islets of G19 compared to virgin rats, a gene that encodes peroxisome proliferator-activated receptor beta protein (**Figure 6D**). The expression of *Pc* which encodes pyruvate carboxylase, that has been shown to be an important islet beta-cell enzyme, was shown to be reduced by 34% in late pregnant rat (G19) compared to virgin rat islets (**Figure 6A**). There were no other significant effects of pregnancy on the expression of the beta-cell genes that were targeted (**Figure 6**).

## Discussion

**Figure 7:**
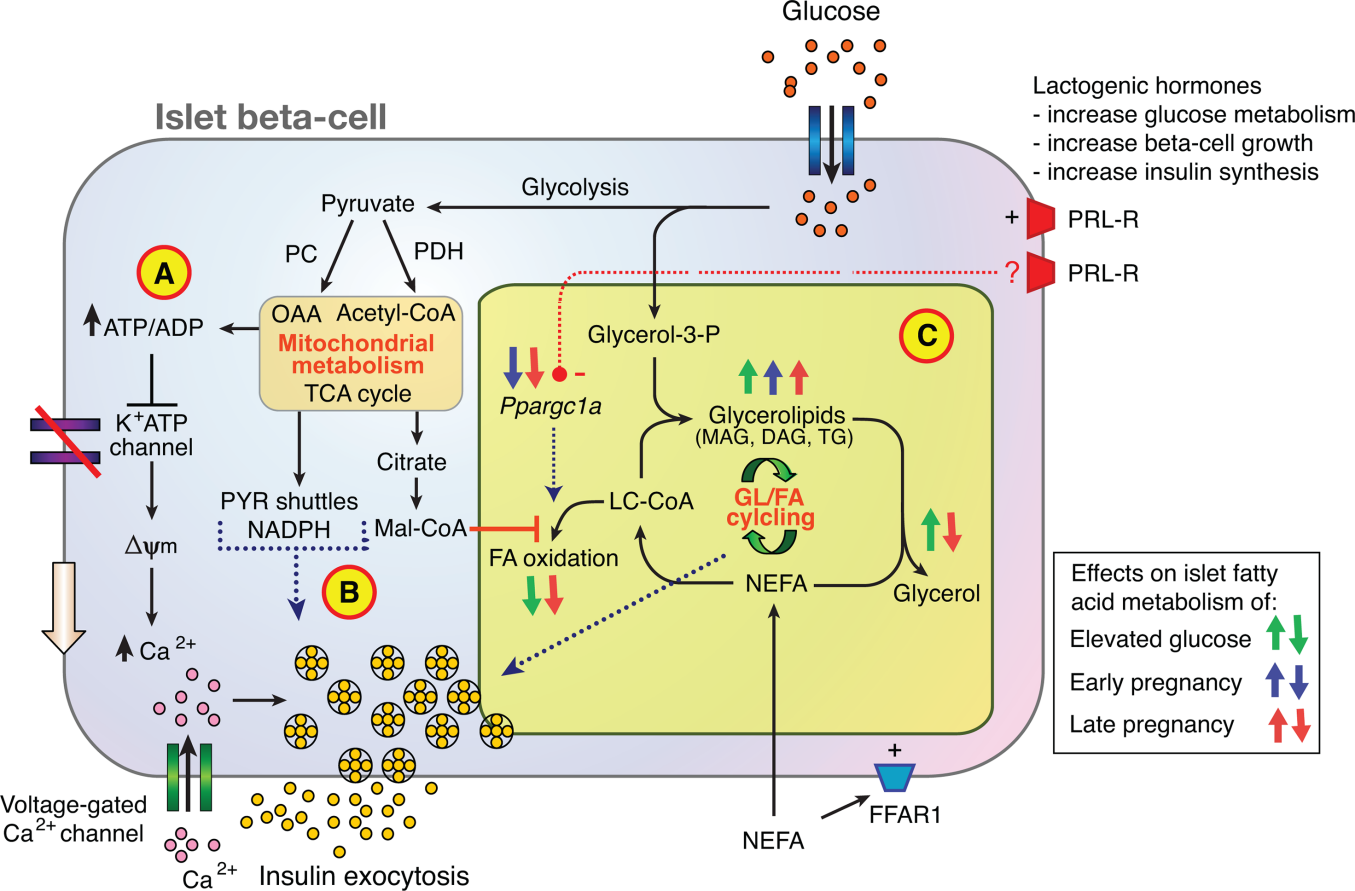
Contribution of glycerolipid/fatty acid cycling (GL/FA) to islet beta-cell adaptation to normal pregnancy. Glucose metabolism promotes insulin secretion *via* **(A)** the K^+^ATP channel dependent triggering pathway, **(B)** the production of mitochondrial-derived anaplerotic-cataplerotic signaling molecules (eg. *via* pyruvate (PYR) shuttles), and **(C)** lipid-signaling molecules such as mono- and diacylglycerols (MAG, DAG) generated by GL/FA cycling. In late rat pregnancy, greater suppression of FA oxidation by glucose, activation of FA esterification processes, and reduced lipolysis to glycerol in islets favor build-up of lipid signaling molecules to amplify insulin secretion. (FFAR1, free fatty acid receptor 1; Mal-CoA, malonyl-CoA; NEFA, non-esterified fatty acid; OAA, oxaloacetate; PC, pyruvate carboxylase; PDH, pyruvate dehydrogenase; PRL-R, prolactin receptor; TCA, tricarboxylic acid).

The results of this study confirm a lowered glucose set-point for insulin secretion in the Sprague-Dawley rat in late pregnancy. Furthermore, glucose-stimulated insulin secretion in isolated islets from pregnant rats is shown to be augmented by fatty acids in association with active GL/FA cycling. Unexpected was a lower TG content in islets of G19 pregnant rats. Of islet lipid metabolism and signaling genes assessed, the expression of the master regulator of mitochondrial metabolism *Ppargc1a* was reduced by 51-64% in islets of pregnant rats (36). Overall, the findings support a role for fatty acid signaling within a multifaceted set of islet beta-cell adaptative mechanisms to pregnancy (**Figure 7**).

All studies were performed in normal chow fed Sprague-Dawley rats which do not develop diabetes. The *in vivo* metabolic status of the rats was first characterized in the fed and fasted states, and by OGTT testing. As the rats were trained to drink the glucose load spontaneously and they were able to freely move around the cage during blood sampling, rat stress was minimized. The combined fetus weights of G19 compared to G11 pregnant rats was 10-fold greater being such that a greater fetal glucose steal likely contributed to the substantially lower fasted and fed blood glucose levels in G19 compared to G11 and virgin rats (37). The lower glycemia achieved through the OGTT in the G19 pregnant rats was also likely a consequence of greater fetal glucose uptake, as well as post-glucose load insulinemia equivalent to that of the virgin rats, despite the lower plasma glucose levels. Thus, the glucose set-point for insulin secretion was lower in the G19 pregnant compared to virgin rats, as reflected in the 53% higher ratio of the AUC_0-120_ of plasma insulin to the AUC_0-120_ plasma glucose following oral glucose. This is consistent with our previous study in Sprague-Dawley pregnant rats (31), and previous reports of a lowered glucose threshold for insulin secretion in pregnancy (8, 17).

As previously reported, serum TG concentrations increased markedly by G19, but were only mildly increased at G11 (24). In fed-rats, serum NEFA trended lower at G11 compared to G19, which although not-significant in this study, has previously been observed (38).

The oscillation in liver glycogen content between the fasted and post glucose-load state was minimal in the virgin rats, but very large in the G19 pregnant rats despite lower glycaemia, indicative of rapid transition from accelerated starvation in which hepatic glycogen is utilised for glucose production to facilitated anabolism in which hepatic glycogen is replenished. These findings are consistent with our previous report of lowering of the maternal glucose homeostasis set-point in the late pregnant rat at the level of the pancreatic islet and liver, with absence of hepatic insulin resistance (31). The results are consistent with previous studies of fasted and fed liver glycogen content in the pregnant rat (39).

Islet protein content was increased in late pregnancy suggestive of increased islet size. Unexpected, however, was the 42% reduction in islet TG content in G19 pregnant compared to virgin rats considering the marked hypertriglyceridemia in late pregnant rats. To our knowledge, the effects of pregnancy on islet TG content have not been previously reported. There are a few possibilities that could explain this finding. First, esterification processes for glycerolipid synthesis are promoted by glucose, such that the lower glycemia of pregnant rats might curtail islet TG synthesis (27). Second lipoprotein lipase activity, responsible for hydrolysis of TG within lipoprotein particles enabling fatty acids to enter cells, is reduced in late pregnancy in many tissues and islets may be involved in this (40). Third, there could be increased activity of TG lipolysis and the TG/FA cycle in late pregnancy, preventing islet TG accumulation (27), to be discussed further below.

The *in vitro* GSIS results support the hypothesis that fatty acid signaling contributes to islet adaptation to pregnancy. As expected, pregnancy did result in increased GSIS, whether expressed per islet, islet protein content, or islet total insulin content. Furthermore, the absolute increase in GSIS, as a result of incubating in the presence of palmitate, expressed per islet protein, was greater in islets of late pregnant rats. The findings are similar to the GSIS results of ZF compared to Zucker lean (ZL) rat islets, in which the effect of palmitate to augment GSIS was markedly enhanced (30). They are also consistent with the effects of fatty acids to augment GSIS in prolactin treated neonatal rat islets (26).

Fatty acids could augment insulin secretion through activating fatty acid receptors, such as Ffar1 (Gpr40), and/or *via* intracellular metabolism with the generation of lipid signaling molecules, such as PL, MAG or DAG from GL/FA cycling. The effects of elevated glucose in the incubation media to increase net islet fatty acid esterification into PL, CE, and TG, to suppress islet fatty acid oxidation, and to activate lipolysis, indicative of glucose-stimulated TG/FA cycling, was evident in all rat groups, as we have previously described outside of pregnancy (27, 30). There was also evidence of pregnancy amplifying components of GL/FA cycling compared to that observed in virgin rats, including net esterification into PL, TG (in G11 and G19 rat islets), as well as MAG (in G11 islets only), and reduced fatty acid oxidation (in G19 islets only). With respect to lipolysis, this was reduced in the G19 compared to G11 rat islets. Other than for PL and TG, the magnitude of changes were small, but considering MAG has been shown to be an important signaling molecule arising from GL/FA cycling for insulin secretion, this finding is of particular interest (41, 42).

The lipolysis findings from G19 rat islets are at variance with those found in islets of the ZF rat, as ZF rat islets had markedly increased rates lipolysis in response to elevated glucose concentrations compared to islets of ZL rats (30). Increased FA esterification, with reduced complete lipolysis to NEFA and glycerol as was found in G19 rat islets, however, would favor a build-up of GL/FA intermediates (e.g. MAG and DAG) which have been shown to have signaling roles in insulin secretion (41–44). Of relevance, the prevention of MAG lipolysis by deletion of the lipase enzyme α/β-hydrolase domain-6 is associated with enhanced insulin secretion (41).

In considering the reduced TG content of G19 islets, taken into context with these fatty acid partitioning results, it is evident that late pregnancy islets are capable of glycerolipid synthesis at 3 mmol/l and 16 mmol/l, as shown for PL and TG. Fatty acid oxidation and lipolysis were reduced in islets from rats late in pregnancy. These adaptations should favor increased rather than reduced TG content. The lower TG content may well be due to the relative hypoglycemia of the late pregnant rat, giving less glucose drive for GL/FA signaling in the basal state. But with a low glucose set-point for glucose uptake and metabolism (45), GL/FA may be promptly activated in the fed state.

Of all islet genes assessed, the reduction in *Ppargc1a* in islets of G11 and G19 pregnant rats was the most obvious change from the non-pregnant state. Furthermore, peroxisome proliferator-activated receptors encoded by *Pparb* and *Ppara*, respectively were lower or trended down, both being regulated by Pparg-coactivator 1-alpha encoded by *Ppargc1a*. Ppargc1a is a transcriptional co-activator important for mitochondrial metabolism, including fatty acid oxidation pathways (36, 46, 47). Prevention of *Ppargc1a* overexpression in islets of type 2 diabetes models or in response to glucolipotoxity improve islet function, yet its total knock out in islet beta-cells causes impaired islet function (46, 47) suggesting complex important roles for this co-activator in islet beta-cells. We have previously shown that *Ppara* knock out results in increased insulin secretion, by inhibiting fatty acid oxidation (33). Thus, the reduction in *Ppargc1a* and *Pparb* may have a role in curtailing islet fatty acid oxidation observed in the islets of pregnant rats found in this study. In support of our findings, prolactin has been shown to reduce *Ppargc1a* expression and expression of other fatty acid oxidation genes in INS1 beta-cells, as well as prevent the effects of dexamethasone to increase beta-cell fatty acid oxidation (48). This may also be a mechanistic pathway by which prolactin treatment of neonatal islet enhanced insulin secretion in the presence of FAs (26).

*Pc* encodes pyruvate carboxylase which is an important enzyme directing glycolysis products into mitochondrial anaplerotic metabolism important in islet beta-cells for normal nutrient-stimulated insulin secretion (29). It seems counter-intuitive for *Pc* expression to be reduced in islets from pregnant rats. Of note *Ffar1* that encodes the long-chain fatty acid receptors was not changed by pregnancy, nor was expression of other key islet beta-cell transcription factor and function genes or other genes regulating islet beta-cell metabolism.

As shown in this and other studies, a lowering of the glucose set-point for insulin secretion in pregnancy is one of the salient characteristics of metabolic adaptation to pregnancy. In considering the potential mechanisms, the results of this study support a role for FA signaling, as a reduction in islet FA oxidation would result in increased availability of FAs for GL/FA cycling and the production of lipid signaling molecules. Islet fatty oxidation is considered an important off-signal for GSIS, that is most important in the fasting state when blood glucose is low (27, 33).

In considering the limitations of the experiments performed, we have not measured insulin clearance which is needed to accurately determine *in vivo* insulin secretion rates. Another major limitation was that *in vitro* islet responses of pregnant rat islets to the presence of fatty acids were assessed in the absence of the normal *in vivo* pregnancy hormonal, metabolic and other humoral factors. For example, the fatty acid oxidation experiments were prepared over 16 h and fatty acid esterification was measured over 16 h, a time in which pregnancy hormonal effects could wear off. The studies were performed using palmitate, which in longer incubations can cause islet (gluco)lipotoxity (34). We previously did not find differences between oleate and palmitate in shorter term incubations on islet function.

**Figure 7** places the new findings of the current study into context of current knowledge on islet beta-cell adaptation to pregnancy. Previous investigators have shown that lactogenic hormone signaling through the prolactin receptor is a major contributor to rodent islet beta-cell adaptation to pregnancy, that promotes beta-cell mass expansion, increased insulin synthesis and enhanced glucose uptake and metabolism (7–9). Glucose metabolism promotes insulin secretion *via* the K^+^ATP channel dependent triggering pathway (**Figure 7A**), the production of mitochondrial-derived anaplerotic-cataplerotic signaling molecules (eg. *via* pyruvate shuttles) (**Figure 7B**), and lipid-signaling molecules such as MAG and DAG generated by GL/FA cycling (**Figure 7C**) (21, 27, 41). The focus of the current study was on the effects of pregnancy on islet fatty acid signaling, including *via* GL/FA cycling (**Figure 7C**). Glucose interacts with NEFA to promote activity in the beta-cell GL/FA cycle by elevating malonyl-CoA which inhibits partitioning of long chain acyl-CoA (LC-CoA) to FA oxidation. LC-CoA are then more available for esterification processes. Glucose also provides the glycerol-3-phosphate necessary for FA esterification into complex lipids. Glycerolipids including TG formed are hydrolysed by lipases, *via* intermediates with signaling capacity (e.g. MAG, DAG) back to the NEFA and glycerol (21, 27, 41). In late rat pregnancy, greater suppression of FA oxidation by glucose, activation of FA esterification processes, and reduced lipolysis to glycerol in islets favor build-up of lipid signaling molecules to amplify insulin secretion. In early pregnancy, the esterification component alone is increased. Early and late pregnancy reduces islet *Ppargc1a* mRNA expression (**Figure 7C**). Ppargc1a is a key transcriptional co-activator important for maintaining FA oxidation. As prolactin has previously been shown to reduce *Ppargc1a* expression and FA oxidation in a beta-cell line (48), lactogenic hormones may, in addition to the mechanisms already established, act to adapt islets to pregnancy *via* up-regulating of GL/FA cycling through down-regulating Ppargc1a. The prolactin receptor signaling processes leading to reduced expression of *Ppargc1a* warrant further investigation. FAs may also enhance insulin secretion *via* the fatty acid receptor (FFAR1) (28), however, expression of this receptor was not shown to be altered in the current experiments.

In conclusion, islet adaptation to pregnancy enabling increased glucose-stimulated insulin secretion was evident in these studies in the absence of fatty acids, indicative of a fatty acid independent component to the adaptation. Islets from pregnant dams, however, were at least as responsive to the effects of fatty acids in augmenting GSIS as virgin rats, such that the mechanisms of fatty acid augmentation of insulin secretion are intact and operating in pregnancy. Further to this, there is some evidence that islets, particularly of late pregnant rats, are more responsive to fatty acid signaling than virgin rats. Pregnancy changes in islet fatty acid partitioning included increased net fatty acid esterification and reduced fatty oxidation, both of which favor increased activity of GL/FA cycling (**Figure 7**). Reduced islet triglyceride content in islets of pregnancy rats was unexpected and warrants further investigation. Reduced expression of *Ppargc1a* in islets of pregnant rats may have a role in the adaptation of islets to pregnancy *via* effects on fatty acid signaling pathways and this, according to previous published work (48), may be regulated by signaling *via* the prolactin receptor. Finally, the lowered set-point for plasma glucose homeostasis in pregnant rats does not support the commonly held view that pancreatic islets during pregnancy adapt to compensate for insulin resistance. Rather, the islets in pregnancy seem to respond to hormonal and metabolic signals with increased insulin secretion to sustain facilitated anabolism, including in response to feeding in the later stages of pregnancy.

## Data Availability Statement

The original contributions presented in the study are included in the article/**Supplementary Material**. Further inquiries can be directed to the corresponding author.

## Ethics Statement

The animal study was reviewed and approved by Royal Melbourne Hospital Animal Ethics Committee and the Australian National University Animal Experimental Ethics Committee (Project F.MS.17.08).

## Author Contributions

CN, JP, and VD-A conceived the study. J-HK, VD-A, JC, DL, and CN performed the experiments and analysed the results. J-HK wrote the first draft of the manuscript, with revisions by VD-A and CN. All authors reviewed the manuscript and contributed to and approved the final draft.

## Funding

This work was supported by grants from the Diabetes Australia Research Program (to CN), the Canberra Hospital Private Practice Fund (to CN) and the National Health and Medical Research Council (Project Grant 418077, to CN).

## Conflict of Interest

The authors declare that the research was conducted in the absence of any commercial or financial relationships that could be construed as a potential conflict of interest.

## Publisher’s Note

All claims expressed in this article are solely those of the authors and do not necessarily represent those of their affiliated organizations, or those of the publisher, the editors and the reviewers. Any product that may be evaluated in this article, or claim that may be made by its manufacturer, is not guaranteed or endorsed by the publisher.
