# Supplementary material for: The Role of Fatty Acid Signaling in Islet Beta-Cell Adaptation to Normal Pregnancy
Source: Front Endocrinol (Lausanne). 2022 Jan 5;12:799081. doi: 10.3389/fendo.2021.799081 (PMC8766493; doi:10.3389/fendo.2021.799081)
Supplement: Supplementary file 1 [file DataSheet_1.docx]

Supplementary Material

**Supplementary Table 1: Real-time PCR Primer Sets.**

| **Gene** | **Forward primer sequence** | **Reverse primer sequence** |
| --- | --- | --- |
| *Acaca* | CAA CCA CTA CGG CAT GAC TCA | AGG TGG TGT GAA GGC GTT GT |
| *Abhd6* | AGG ATA TGT GGC TCA GTG TGG | GTG CCT ATA AGG TGA AAG GGC |
| *Acox1* | CCA CTA CGT GGT CGT TAA GGT CTT | GGA CGG CTT TGT CTT GAA TCT T |
| *Pnpla3* | TGC CCG AAT GAC CAT GTG | GGT TAT CCT TGG ATG CTC TGG AT |
| *Arnt* | ACA TCC ACT GAC GGC TCC TAC | ACA TAC ACC ACT CGG CCA GTC |
| *Actb* | CGT GAA AAG ATG ACG CAG ATC A | CAC AGC CTG GAT GGC TAC GT |
| *Cpt1* | GCA TCG ACC GCC ATC TCT | CTG CAA GAT ACT TGG ACA CCA CA |
| *Fasn* | CAG CTG TCA GTG TAA AGA AAC ATG TC | AGC TCA CGT GCA GTT TAA TTG TG |
| *Gck* | TCT GTC ACC GAC TGC GAC AT | CGC GAG TGG ACA CGC TTT |
| *Slc2a2* | CTG TCT GTG TCC AGC TTT GCA | CAA GCC ACC CAC CAA AGA AC |
| *Gipr* | GAT GGA TGG ACA CAC AGA CAC | AGA GTC TCC ACT CCA GAA GGC |
| *Glp1r* | TGA GAG ACC TGC CCT TGG AAC | CGA GAG GAA GGC TGA TGT AGG |
| *Gpam* | CCT GTG GGC ATC TCG TAT GA | GCC CAA CTG TTC ACC ATT GTA G |
| *Ffar1* | TTA GCC ATC CGA GGT GCA GTG | GAA GCC TCC ACC TGC ATA GAG |
| *Gpr119* | GGT TGT GGC TAT GCT GCT ATC | TTC TGT GTG TGC TGA GCA GAG |
| *Hif1a* | CAG GAT GGA ATG GAG CAG AAG | ATC GTA ACT GGT CAG CTG TGG |
| *Lipe* | AGA CGG GCC TCA GTG TGA CT | AGA CGG GCC TCA GTG TGA CT |
| *Ins2* | CTG CCC AGG CTT TTG TCA A | TTC CAC CAA GTG AGA ACC ACA |
| *Mgll* | TTG AAG AGG CTG GAC ATG CTG | TAG TCC TTC TGG ACG GTG TTC |
| *Pc* | AAT TGC AGA GGA GTT TGA GGT TG | GGG CTT TGA TGT GCA AGG TC |
| *Pdx1* | GAA CCG GAG GAG AAT AAG AGG | AGT CAA GTT GAG CAT CAC TGC |
| *Ppargc1a* | ACC GCA CAC ATC GCA ATT C | CGG CTG TAG GGT GAC CTT GA |
| *Ppara* | TCG GCG AAC TAT TCG GCT AA | CAG CTT CGA TCA CAC TTG TCG T |
| *Pparb* | AAG AAC ATC CCC AAC TTC AGC A | CAG GGT CAC CTG GTC GTT GA |
| *Pparg* | TGA CTT GGC CAT ATT TAT AGC TGT CA | CGA TGG GCT TCA CGT TCA G |
| *Scd* | CCC ACA TGC TCC AAG AGA TCT C | GGA AGG AGG CTC TGT GAT GGT |
| *Srebf1* | AGC ACA GCA ACC AGA AAC TCA A | AGG TCT TTC AGT GAT TTG CTT TTG T |
| *Ucp2* | TGA GCC TCT CCA GCT GAT GA | CTG GGC AGA GGA TGA AGG AA |

**Supplementary Figure 1.** Insulin secretion in isolated pancreatic islets from virgin (V), gestational age 11 day (G11) and gestational day 19 (G19) rats. Static insulin secretion at glucose concentrations of 3 (3G), 8 (8G) and 16 (16G) mmol/l in the absence or presence of 0.25 mmol/l BSA-bound palmitate (+FA), expressed as secretion per islet **(A)**, and expressed as secretion as a % total insulin content expressed. Means ± SEM; three-way ANOVA (A and B); multiple comparisons, G11 vs virgin not significant, G19 vs virgin *P*<0.001, G19 vs G11 *P*<0.005 (A); G11 vs virgin *P*<0.005, G19 vs virgin not significant, G19 vs G11, *P*<0.05 (B).
